# The prevalence of common mental disorders among hospital physicians and their association with self-reported work ability: a cross-sectional study

**DOI:** 10.1186/1472-6963-12-292

**Published:** 2012-08-31

**Authors:** Martijn M Ruitenburg, Monique HW Frings-Dresen, Judith K Sluiter

**Affiliations:** 1 Coronel Institute of Occupational Health, Academic Medical Center, University of Amsterdam, PO Box 22700, 1100, DE Amsterdam, the Netherlands

## Abstract

**Background:**

We studied the prevalence of common mental disorders among Dutch hospital physicians and investigated whether the presence of a mental disorder was associated with insufficient self-reported work ability.

**Methods:**

A questionnaire was sent to all (n = 958) hospital physicians of one academic medical center, using validated scales to assess burnout, work-related fatigue, stress, posttraumatic stress disorder (PTSD), anxiety and depression. Furthermore, respondents were asked to rate their current work ability against the work ability in their own best period (adapted version of the first WAI item). The prevalence of each common mental disorder was calculated. In addition, odds ratios of reporting insufficient work ability for subjects with high complaint scores compared to physicians with low complaint scores were calculated for each mental disorder.

**Results:**

The response rate was 51%, and 423 questionnaires were eligible for analysis. The mental disorder prevalence rates were as follows: work-related fatigue 42%, depression 29%, anxiety 24%, posttraumatic stress complaints 15%, stress complaints 15% and burnout 6%. The mean score for self-reported work ability was 8.1 (range 0–10), and 4% of respondents rated their own work ability as insufficient. Physicians with high mental health complaints were 3.5- for fatigue, 5.6- for PTSD, 7.1- for anxiety, 9.5- for burnout, 10.8- for depression and 13.6-fold for stress more likely to report their work ability as insufficient.

**Conclusions:**

The prevalence of common mental disorders among hospital physicians varied from 6% for burnout to 42% for work-related fatigue. Those physicians with high complaints had significantly 4- to 14 times increased odds of reporting their own work ability as insufficient. This work suggests that to ensure future workers health and patients safety occupational health services should plan appropriate intervention strategies.

## Background

The number of vacant jobs for hospital physicians in the Netherlands reached its highest point ever near the end of 2009 [1]. Due to the aging society, the number of patients and the number of chronic diseases will increase and, subsequently, so will the required care and number of hospital physicians. In addition, a great exodus of physicians will take place as many reach their retirement age [2]. To keep health care availability at the desired level, it is important to keep hospital physicians healthy in their job and to prevent ill health or even absenteeism.

Work-related fatigue has been associated with increased sick leave [3]. A more severe form of fatigue is burnout. As another aspect of physicians’ psychological health, burnout increases the occurrence of sickness and absenteeism, and it is an example of a mental disorder that occurs in physicians, as reviewed in Sanderson and Andrews [4] and De Valk and Werner [5].

Physicians’ mental health is also important because it can affect their work performance and thereby patient safety [6]. For example, depressive symptoms among physicians have been reported to lead to medical mistakes and to adversely affect physicians’ attitudes towards patients; residents with burnout have reported significantly more errors than those not suffering from burnout [7-9]. In addition, moderate and high psychological distress increases the odds for workplace failure and decreases the odds for workplace success [10]. Mental disorders not only affect work performance but also influence other aspects of work, such as the quality of interaction with patients and colleagues. Negative aspects of mental well-being, such as burnout, may be associated with a lower capacity for empathy and suboptimal patient care [11-13]. Prolonged fatigue has been found to be negatively associated with the relationship quality between physicians and staff [14]. Together, these findings indicate that the mental health of hospital physicians influences their job performance, which is reflected in both the risk of making mistakes and in the quality of interaction with patients and colleagues. Job performance can be examined using the concept of work ability, as measured by the Work Ability Index (WAI) [15]. Work ability is a measure of the degree to which a worker is physically and mentally able to cope with the demands at work [15]. Decreased work ability is associated with reduced job performance and with an increased risk of long-term sickness absence [16,17]. It would be interesting to investigate the level of work ability in hospital physicians and the relationship with the presence of a common mental disorder.

Several studies conducted outside the Netherlands have indicated that physicians are at risk of developing common mental disorders and that these have an impact on their quality of work. These studies either investigated the relationship between general well-being and quality of work or focussed on an aspect of mental health, like depressive symptoms, and its association with quality of work [7,11,12]. In the Netherlands, some studies have examined hospital physicians and mental disorders, but these studies either only focused on burnout [9,18,19] or did not report any prevalence rates [20]. To our knowledge, no study has evaluated the prevalence among working hospital physicians of the most common mental disorders found in the general working population. Therefore, this study had two aims: to investigate the prevalence of six common mental disorders among one population of Dutch hospital physicians (i.e., work-related fatigue, stress, depression, anxiety, burnout and posttraumatic stress disorder) and to investigate whether the presence of a mental disorder is associated with the way physicians perceive their own work ability.

## Methods

### Population and procedure

All 958 medical doctors working in one academic medical center in the Netherlands were eligible to participate in this study. This population included all specialists in 1 of the 23 subspecialties and all medical residents, doctors following a specialisation specialty after graduating from university, working in one academic medical center in the Netherlands. In autumn of 2009, the participants received an email with information about the study followed by an email with a link and a personal password to an online questionnaire. This questionnaire was part of a larger study to gather information to develop a job-specific workers’ health surveillance programme for hospital physicians in all academic medical centers in the Netherlands. The participants were asked to fill out the questionnaire within two months after receiving the email. Participants gave their informed consent to participate by starting the online questionnaire. For performing this cross-sectional study no official medical ethical procedure is obliged in the Netherlands.

### Study measures

The questionnaire contained items to gather general sample information concerning age, gender and seniority (physician or resident). Furthermore, data were gathered concerning work-related psychological health effects including burnout, posttraumatic stress disorder (PTSD), work-related fatigue, stress, depression and anxiety.

Burnout was measured using the Dutch version of the Maslach Burnout Inventory (MBI) [21]. Burnout encompasses three domains: emotional exhaustion, depersonalisation and personal accomplishment. Following Maslach et al. [21], the domains of emotional exhaustion (eight items) and depersonalisation (five items) were used for this study. These thirteen items are scored on a seven-point frequency scale from 0–6. Each scale score is computed by summing the scores of the items, resulting in a range for emotional exhaustion of 0–48 and a range for depersonalisation of 0–30.

PTSD was measured using the Dutch version of the Impact of Event Scale [22]; Dutch version, [23]. The translated version showed good reliability and construct validity [24]. The scale consists of fifteen items scored on a four-point frequency scale from 0–5. Participants are asked to rate how frequently during the last seven days they experienced certain thoughts and feelings related to a particular life event, for example the death of a patient (e.g., ‘I had waves of strong feelings about it’). A scale score is computed by summing the scores on each item, resulting in a scale score ranging from 0 to 75.

Work-related fatigue was measured using the need for recovery after work scale from the Dutch Questionnaire on the Experience and Evaluation of Work [25]. This scale contains eleven statements using a yes/no format. An example is as follows: ‘generally, I need more than an hour before I feel completely recuperated after work’. The scale scores range from 0–100.

Psychological distress complaints were measured using the distress screener, which is a shortened version of the four-dimensional symptom questionnaire (4DSQ) [26]; original version, [27]. This shortened version consists of three items scored on a three-point frequency scale from 0–2. The scores of the items are summed to obtain a scale score.

Both depression and anxiety were measured with their respective subscales of the Brief Symptom Inventory (BSI) [28], the abbreviated version of the Dutch version of the Symptom Checklist-90 (SCL-90) [29]. These scales consist of six items that are scored on a five-point frequency scale from 0–4. The scores on the separate items are summed and divided by the total number of items, resulting in scale scores ranging from 0–4.

### Work ability

Using an adapted, profession-specific version of the first question of the Work Ability Index (WAI) [15], physicians were asked to rate their own current work ability against their work ability as a physician in their own best period on an eleven-point scale from 0–10, with ten being the best work ability in their own best period of life. Subjects reporting a work ability score lower than six are considered to rate their own work ability as insufficient (in analogy with the school rating system in the Netherlands).

### Analyses

The study population’s mean scores and frequencies for the demographic data were calculated first. Mean scores were calculated for each common mental health disorder. In addition, based on established cut-offs for each complaint, two groups were composed of subjects with and without a common mental disorder. The prevalence for each mental disorder was calculated. A case of burnout was defined as an individual with high scores for both emotional exhaustion (score ≥27) and depersonalisation (score ≥10) [21]. For PTSD, the cut-off score of 26 or higher was used because people are then suspected to suffer from PTSD [30]. For work-related fatigue, people with scores higher than 54.5 are considered to have high work-related fatigue [31]. With a scale score ≥4 on the distress screener, a subject is considered to have high stress complaints [26]. For both depression and anxiety, subjects with a score ≥0.41 are considered to have high complaints [28]. Mean scores were calculated for the self-reported work ability and the percentage of participants rating their own work ability as either sufficient or insufficient.

To investigate the relationship between the occurrence of health complaints and self-reported work ability, the odds ratio of reporting insufficient work ability for subjects with high complaints was estimated for each psychological health complaint and compared to those with low complaints. This was done by performing a binary logistic regression analysis. Analyses were performed using SPSS 17.0 for Windows.

## Results

There was a total of 458 survey respondents (51%); 29 questionnaires were not analysed because these respondents had no clinical duties and had primarily managerial duties. Six questionnaires were lost due to incomplete responses. A final number of 423 questionnaires were used for the analysis.

### Demographic variables

Table 1 shows selected characteristics of our study population. A little more than half of the respondents were working as medical doctors (54%). A slight majority of respondents were female (53%), mainly due to a greater number of female respondents among the medical residents (58%). The age of the total population averaged 41 years (SD = 10.1); the medical doctors (mean age = 47 years, SD = 8.9) were significantly older (t = 21.73, p < .05) than the medical residents (mean age = 33 years, SD = 3.2).

**Table 1:** Overview of the demographic characteristics of the study population

	**Total**		**Medical resident**		**Medical doctor**	
			**[46%]**		**[54%]**	
	***%***	***[n]***	***%***	***[n]***	***%***	***[n]***
Male	47	[199]	42	[80]	52	[119]
Female	53	[223]	58	[113]	48	[110]
	***mean***	***[sd]***	***mean***	***[sd]***	***mean***	***[sd]***
Age [years]	41	[10.1]	33	[3.2]	47	[8.9]

### Prevalence of common mental health disorders

An overview of the mean scores and prevalence rates of the examined common mental health disorders for subgroups of gender, profession and age is presented in Table 2.

**Table 2:** Mean score values and the percentage [%] of participants scoring high on each health complaint

	**Total**	**Gender**		**Profession**		**Age [years]**			
		***Male***	***Female***	***Medical doctor***	***Medical resident***	***20-35***	***36-45***	***46-55***	***> 56***
**Burnout**	[n = 395]	[n = 187]	[n = 207]	[n = 214]	[n = 181]	[n = 168]	[n = 115]	[n = 63]	[n = 49]
Emotional exhaustion [mean; 0–48]	14.1	12.5	15.5	13.3	15.0	14.8	13.6	13.9	12.8
SD	8.0	7.2	8.5	8.0	8.0	14.8	13.6	8.8	7.3
Depersonalisation [mean; 0–30]	5.4	5.4	5.3	4.5	6.4	6.0	5.5	4.8	3.6
SD	4.4	4.4	4.4	4.1	4.5	4.2	4.9	4.6	3.3
Burnout indicative	6%	3%	9%	6%	7%	7%	5%	10%	2%
**PTSD**	[n = 380]	[n = 178]	[n = 201]	[n = 206]	[n = 174]	[n = 164]	[n = 109]	[n = 60]	[n = 47]
mean [0–75]	10.6	8.3	12.8	11.0	10.1	10.4	10.6	11.6	10.5
SD	13.1	11.6	14.0	13.4	12.8	12.5	13.3	14.7	12.8
PTSD indicative [≥ 26]	15%	9%	20%	16%	14%	13%	17%	15%	15%
**Work-related fatigue**	[n = 389]	[n = 182]	[n = 206]	[n = 211]	[n = 178]	[n = 166]	[n = 112]	[n = 63]	[n = 48]
mean [0–100]	44.3	39.2	49.0	41.1	48.0	47.6	38.6	44.4	45.8
SD	29.2	29.0	28.6	28.3	29.8	29.2	29.1	28.3	29.2
high [>54.5]	42%	36%	49%	37%	48%	48%	35%	40%	44%
**Stress complaints**	[n = 398]	[n = 186]	[n = 211]	[n = 216]	[n = 182]	[n = 169]	[n = 116]	[n = 63]	[n = 50]
mean [0–6]	1.9	1.6	2.2	1.7	2.1	2.1	1.7	1.8	1.6
SD	1.7	1.5	1.8	1.6	1.8	1.8	1.5	1.9	1.5
high [≥ 4]	15%	9%	20%	12%	19%	18%	11%	14%	12%
**Depression**	[n = 400]	[n = 189]	[n = 210]	[n = 216]	[n = 184]	[n = 170]	[n = 116]	[n = 64]	[n = 50]
mean [0–4]	0.35	0.29	0.41	0.32	0.39	0.36	0.35	0.37	0.32
SD	0.5	0.4	0.6	0.5	0.6	0.6	0.5	0.5	0.5
Depression indicative	29%	25%	32%	27%	31%	29%	27%	30%	34%
[>0.41]									
**Anxiety**	[n = 390]	[n = 187]	[n = 202]	[n = 211]	[n = 179]	[n = 165]	[n = 114]	[n = 63]	[n = 48]
mean [0–4]	0.33	0.25	0.40	0.27	0.39	0.39	0.27	0.35	0.22
SD	0.5	0.4	0.6	0.4	0.6	0.5	0.4	0.6	0.3
Anxiety indicative	24%	18%	29%	19%	30%	30%	17%	24%	19%
[>0.41]									

Two out every five physicians (42%) reported high work-related fatigue. One quarter of physicians were found to have high depression (29%) and anxiety (24%) complaints, and one out of every six (15%) physicians had high PTSD and stress complaints. The prevalence of burnout was 6%.

Within the subgroups of gender, profession and age, some variation was observed in the prevalence rates of high scores for mental health disorders (Table 2). For example, the prevalence rate of female physicians with a (posttraumatic) stress-indicative score (20%) was twice as high as male physicians (9%).

### Work ability and the relationship with each psychological health complaint

As shown in Table 3, hospital physicians had a mean work ability score of 8.1, and 4% of physicians rated their work ability as insufficient.

**Table 3:** Ratings of self-reported work ability and number of subjects with insufficient or sufficient work ability

	**Total**	**Gender**		**Profession**		**Age [years]**			
		***male***	***female***	***medical doctor***	***medical resident***	***20-35***	***36-45***	***46-55***	***> 56***
	**[n = 405]**	**[n = 191]**	**[n = 213]**	**[n = 219]**	**[n = 186]**	**[n = 171]**	**[n = 119]**	**[n = 65]**	**[n = 50]**
	***mean***	***mean***	***mean***	***mean***	***mean***	***mean***	***mean***	***mean***	***mean***
Work ability	8.1	8.4	7.8	8.2	7.9	7.9	8.3	8.3	7.8
	*SD*	*SD*	*SD*	*SD*	*SD*	*SD*	*SD*	*SD*	*SD*
	1.5	1.4	1.5	1.5	1.4	1.6	1.2	1.2	1.8
	*%*	*%*	*%*	*%*	*%*	*%*	*%*	*%*	*%*
Insufficient [score < 6]	4	2	5	3	4	5	3	2	4
Sufficient [score ≥ 6]	96	98	95	97	96	95	97	98	96

Table 4 shows the results from the analyses in which each psychological health complaint was examined in relation to insufficient work ability. These analyses showed that physicians with scores indicative of mental health disorders were 3.5- for fatigue, 5.6- for PTSD, 7.1- for anxiety, 9.5- for burnout, 10.8- for depression and 13.6-fold for stress more likely to report their work ability as insufficient than those without scores indicative of mental health disorders.

**Table 4:** Overview of the odds ratios [ORs] for insufficient work ability by high psychological health complaints

	***[n]***	***OR***	***95% CI***	***Sig.***
**Burnout**				
low	[371]			
high	[24]	9.5	3.0 - 30.6	0.000
**PTSD**				
low	[324]			
high [≥ 26]	[56]	5.6	2.0 – 16.3	0.001
**Work-related fatigue**				
low	[224]			
high [>54.5]	[165]	3.5	1.1 – 11.5	0.035
**Stress complaints**				
low	[339]			
high [≥ 4]	[59]	13.6	4.5 – 41.6	0.000
**Depression**				
low	[284]			
high [> 0.41]	[116]	10.8	3.0 – 39.1	0.000
**Anxiety**				
low	[298]			
high [> 0.41]	[92]	7.1	2.4 – 21.5	0.000

## Discussion

To our knowledge, this is the first study to investigate the prevalence of several relevant common mental disorders among one group of hospital physicians and the association of these disorders with self-reported work ability. We found prevalence rates of 42% for work-related fatigue, 29% for depression, 24% for anxiety, 15% for PTSD and stress and 6% for burnout. Physicians rated their own current work ability with a mean score of 8.1 (range 0–10), and 4% of physicians rated their current work ability as insufficient, indicating that they were having difficulties coping with the demands of their work. Physicians with high mental health disorder scores were 3.5- to 13.6-fold more likely to report their work ability as insufficient compared to colleagues without those complaints.

The relatively high prevalence rates of the common mental disorders might theoretically be affected by the potential correlation between the investigated psychological variables. Post-hoc analysis revealed that the explained variance between the raw scores of any two psychological complaints scales did not exceed 50% in our study. We only used validated scales with established cut-off points to assess the prevalence rates of the investigated common mental disorders. In addition, missing data about the mental health status of the non-responding physicians might lead one to argue that the prevalence rates of common mental disorders in the total population might differ from the ones found in this study. When performing a theoretical exercise, one might reason that the prevalence estimates for each of the mental health complaints would decrease to half of the reported prevalence rates when the other 49% of the hospital physicians would not report any complaints. On the other hand, when one reasons that all of the other 49% of hospital physicians would at least report one mental health complaint, the prevalence rates would increase dramatically. Variation on several factors in this population seems to make it impossible to predict in which direction this bias would go. However, although this might affect the reported prevalence, it seems unlikely that the relationship between the presence of a mental health complaint and self-reported work ability would be significantly affected.

No other studies were found investigating the prevalence rates of work-related fatigue and stress among hospital physicians, indicating that knowledge on this subject is both relevant and needed. In comparison with other studies reporting prevalence rates for burnout of 13% and 21% among residents in the Netherlands [18,19], this study found a relatively low prevalence of burnout (6%). However, these studies also reported that 4-6% of the medical residents had severe burnout, which seems more in line with our definition. Studies conducted outside the Netherlands reported that 22-24% of hospital physicians were labelled as cases of burnout [32,33]. These differences might be associated with differences in work demands [e.g., working hours]. Compared to prevalence rates for depression found in other studies, which varied between 8% and 29% [34-37], we found a relatively high percentage of physicians (29%) with depressive symptoms indicative of a depressive disorder. The percentage of physicians reporting PTSD (15%) was similar to that found in one other study [38], but it was larger than the 4% found in a study among Canadian physicians [39]. The prevalence rate for anxiety complaints (24%) was similar to the prevalence rates of anxiety disorders of 17% in men and 26% in women found in another recent study [36].

In this cross-sectional study it was found that hospital physicians with psychological ill health were significantly more likely to report insufficient work ability compared to hospital physicians with good mental health. The number of hospital physicians (4%) reporting insufficient work ability is considered relatively high. Hospital physicians rating their own work ability as insufficient might be at risk for future sickness absence because reduced self-reported work ability has been strongly associated with an increased risk of long-term sickness absence [17]. Reduced work ability has also been associated with reduced job performance [16]. Therefore, reduced work ability and ill mental health may result in hospital physicians putting patient safety at risk. This idea is supported by several studies showing that mental health complaints, such as burnout and depression, increase the risk for medical mistakes and workplace failure [7,10,40].

In the Netherlands, several attempts have been made to improve and ensure patient safety, varying from introducing changes at the organisational level of hospitals to improving clinical technologies. For example, a new surgical safety checklist has been developed to reduce the number of surgical complications [41]. From the perspective of occupational health, our findings indicate that more attention should be given to the mental health of hospital physicians to ensure patient safety. Using a job-specific workers’ health surveillance programme, the mental health conditions of physicians can be monitored to identify physicians showing early signs of developing a common mental disorder and to intervene accordingly to decrease these complaints. In addition to focusing on health effects, the exposure to certain job characteristics of hospital physicians should also be considered. Current literature indicates that the work of hospital physicians puts them at risk of developing common mental disorders. In general, job strain and high psychological demands, particularly prevalent in the work of hospital physicians, can predict common mental disorders [14,42]. Physicians may be vulnerable to developing depressive symptoms because of their everyday exposure to patient suffering, disease, death, emergency and unreasonable patient demands [34,43,44]. The long working hours, especially prevalent during residency, put hospital physicians at risk of becoming fatigued [45]. Because physicians are often reluctant to seek help, perhaps because they believe they can help themselves, they are more vulnerable than the general population [43,44]. Thus, it seems reasonable to assume that changes in work conditions can improve physicians’ mental health and subsequently ensure patient safety. However, because some specific job demands in the work of physicians cannot be changed, it seems more beneficial to focus on monitoring the mental health of physicians to capture those at increased risk of decreased work functioning or future sick leave absence. One way to monitor the mental health of physicians is to investigate whether they have high mental health complaints. Based on the strong association found in this study between physicians’ ill mental health and reduced work ability, it may be possible to monitor the mental health of physicians by repeatedly surveying their own current work ability. Reduced self-reported work ability might be a signal of underlying ill mental health, which has been associated with an increased risk of long-term sick leave absence. To maintain physician health on the job, a workers’ health surveillance programme should be established as an occupational health strategy; interventions for high-risk groups of physicians should be used to restore a physician’s reduced work ability and to evaluate the effects of this strategy.

## Conclusions

The prevalence of common mental disorders among hospital physicians varied from 6% for burnout to 42% for work-related fatigue. The mean score for self-reported work ability was 8 (range 0–10) and 4% of the physicians rated their own work ability as insufficient. Those hospital physicians with high complaints had significantly increased odds (between 4 and 14 fold) of reporting their own workability as insufficient. A reduced work ability increases the risk of long term sickness absence and is associated with reduced job performance [16,17]. In order to ensure patients’ safety and to keep physicians healthy at the job, more attention should be given to the mental health of hospital physicians. Monitoring the mental health status of hospital physicians by self-rating of their own current work ability contributes to capturing those physicians at increased risk of decreased work functioning or future sick leave absence.

## Competing interests

The authors declare that they have no competing interests.

## Authors’ contributions

MMR was involved in acquisition, analysis and interpretation of data, and writing of the manuscript. MFD was involved in study design and revising the manuscript. JKS was involved in study design, analysis and interpretation of data and revising the manuscript. All authors have read and approved the final manuscript. JKS and MFD obtained the funding for this study and acted as co-principal investigators. All authors read and approved the final manuscript.

## Pre-publication history

The pre-publication history for this paper can be accessed here:

http://www.biomedcentral.com/1472-6963/12/292/prepub
